# Rab25 GTPase: Functional roles in cancer

**DOI:** 10.18632/oncotarget.19571

**Published:** 2017-07-26

**Authors:** Sisi Wang, Chunhong Hu, Fang Wu, Shasha He

**Affiliations:** ^1^ Department of Oncology, The Second Xiangya Hospital, Central South University, Changsha, Hunan, China

**Keywords:** Rab25, small GTPase, vesicle trafficking, ovarian cancer, renal cancer

## Abstract

Rab25, a small GTPase belongs to the Rab protein family, has a pivotal role in cancer pathophysiology. Rab25 governs cell-surface receptors recycling and cellular signaling pathways activation, allowing it to control a diverse range of cellular functions, including cell proliferation, cell motility and cell death. Aberrant expression of Rab25 was linked to cancer development. Majority of research findings revealed that Rab25 is an oncogene. Elevated expression of Rab25 was correlated with poor prognosis and aggressiveness of renal, lung, breast, ovarian and other cancers. However, tumor suppressor function of Rab25 was reported in several cancers, such as colorectal cancer, indicating the tumor type-specific function of Rab25. In this review, we recapitulate the current knowledge of Rab25 in cancer development and therapy.

## INTRODUCTION

Rab25 (Ras-related protein Rab-25) belongs to the Rab protein family. Until now, 70 Homo sapiens Rab proteins have been identified [1, 2] and they form the largest branch of the Ras superfamily [3]. Rabs are evolutionary conserved and their homologs are found in organisms ranging from yeast to human. Rabs are small GTPases involved in the control of vesicle trafficking. They govern correct transport of substances between different cellular compartments and have been implicated in various cellular functions, including cell proliferation, cell mobility, signal transduction and protein transport [4, 5]. Since they are key regulators of major cellular functions, some of them have been reported to be involved in tumorigenesis and cancer progression. For example, Rab5a and Rab7 were both up-regulated and have a higher degree of membrane association in autonomous thyroid adenomas [6].

Each Rab protein has different subcellular localization and controls specific membrane transport pathways [7–9]. Rab25 belongs to the Rab11 subfamily (Rab11a, Rab11b, and Rab25) that regulates apical transport and/or recycle of vesicles to the plasma membrane [10, 11]. Some Rab proteins are ubiquitously expressed in human tissues, whereas Rab25 is specifically expressed in epithelial cells. Rab25 functions either as an oncogene or a tumor suppressor with a cancer type-dependent manner. It was predominantly reported to have oncogenic function and elevated expression in cancer, including ovarian cancer [12, 13], breast cancer [12–14], renal cancer [15, 16], gastric cancer [17], liver cancer [18], non-small cell lung cancer [19], bladder cancer [20], glioblastoma multiforme [21] and prostate cancer [22]. Tumor suppressor function of Rab25 has also been found in several types of cancer, including colon cancer [23], head and neck cancer [24], esophageal squamous cell carcinoma [25] and oral and oropharyngeal squamous cell carcinoma [26]. The function of Rab25 in cancer is, therefore, multifactorial and tumor type-specific.

This review recapitulates our current knowledge of the role of Rab25 in cancer, including the involvement of Rab25 in cancer prognosis, cancer progression, functional mechanisms and future research directions.

### Structure of Rab25

#### Structure and biological function of Rab25

Rab25 is a 23kDa small GTPase with general GTPase tertiary structure, a central barrel composed of a six-stranded b-sheet surrounded by a-helixs [27]. The control of vesicle trafficking is the major function of Rab proteins and was first reported by Salminen *et al*. [28]. The function of Rab25 is determined by two structural features, the guanine nucleotide binding motif and the carboxyl-terminus region. Guanine nucleotide binding motif allows Rab25 to bind with GTP/GDP, which controls the activity of Rab25. The carboxyl-terminus of Rab25 contains CCXXX motif. Post-translational modification of the C-terminus determines the binding of Rab25 on specific vesicles and regulates the entire membrane trafficking process [4, 29].

Each Rab protein recognizes and controls the transportation of different membrane vesicles. The C-terminus of Rab proteins contain XXXCC, XXCCX, XCCXX, CCXXX or XXCXC motif. Prenylation of the two cysteine residues at the motif, together with the addition of geranylgeranyl groups to one or two cysteine residues near the C-terminus, is required for tight binding of Rab proteins to their specific vesicles [30–32]. Post-translational modifications of the CCXXX motif of Rab25 allow the protein to bind to apical recycling endosomes and regulate recycle of the vesicles to the apical plasma membrane [11].

**Figure 1:**
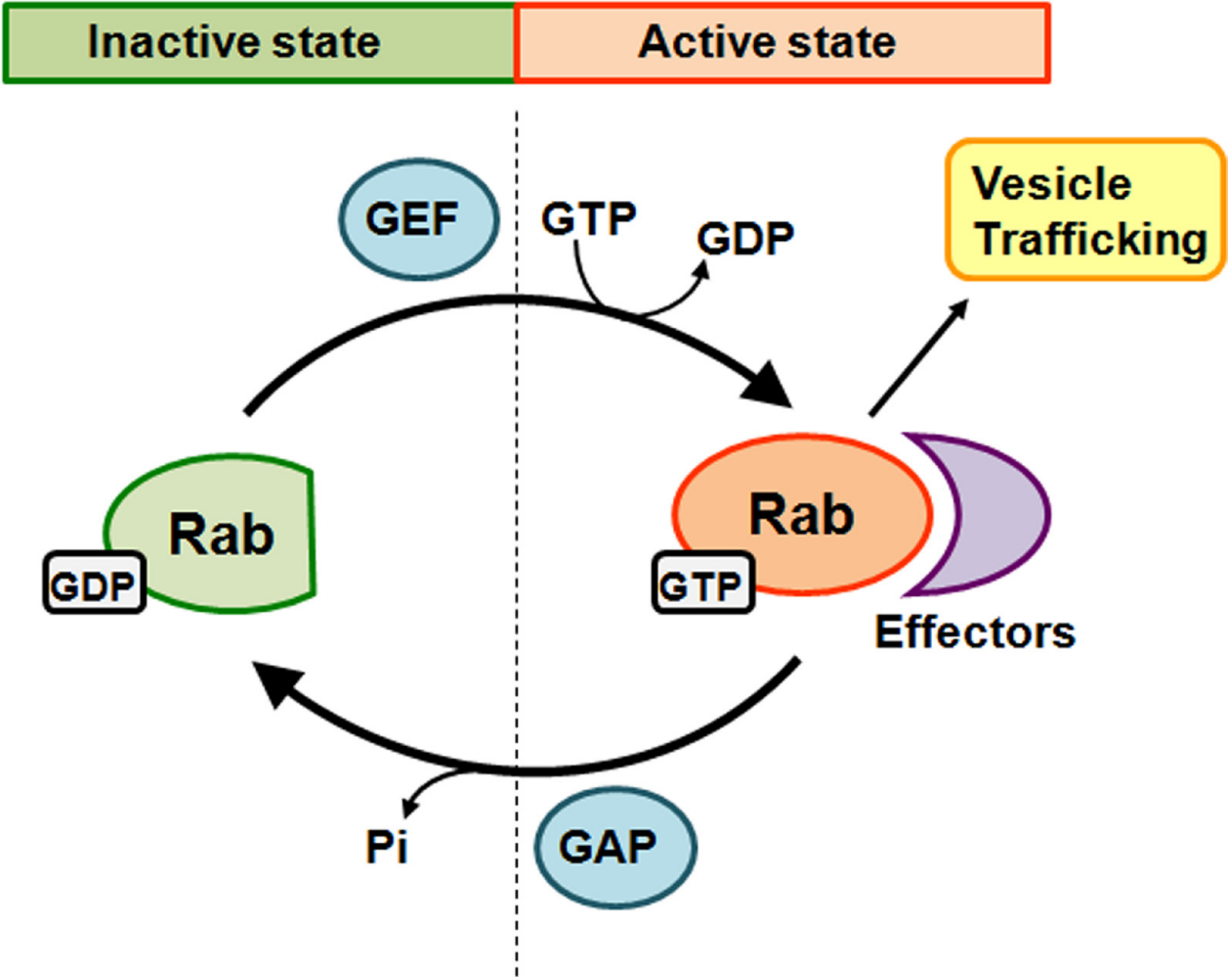
Schematic diagram of Rab GTPase cycle Rabs switch between two conformations, an inactive GDP-bound form and an active GTP-bound form. A guanine nucleotide exchange factor (GEF) catalyzes the conversion from GDP-bound to GTP-bound and leads to activation of Rab. Activated Rab binds with specific effectors to promote vesicle trafficking. The inherent GTP hydrolysis of Rabs together with the enhancing effect of GTPase-activating protein (GAP) leads to Rab inactivation. Conversion of Rabs to the inactive state leads to effector dissociation from the Rab protein.

The Rab proteins cycle between two states, the GTP-bound active state and the GDP-bound inactive state (Figure 1). The inactive Rab proteins located in the cytosol and are recognized by guanine nucleotide exchange factor (GEF), which catalyzes the exchange of GDP for GTP at the guanine nucleotide binding motif to form the active Rab proteins [33]. After coordinating the transport of vesicles to their destination, the active Rab proteins are converted back to the GDP-bound inactive form through hydrolysis of GTP, which is stimulated by a GTPase-activating protein (GAP) [34].

The active GTP-bound Rab protein coordinates the entire 5-step vesicle trafficking process, including vesicle sorting, uncoating, motility, tethering and fusion, by recruiting effector proteins for distinct membrane trafficking steps [35]. GTP-bound Rab proteins activate sorting adaptor to sort a receptor into a budding vesicle [36]. The active Rab proteins promote the vesicle uncoating through the recruitment of phosphoinostide (PI) kinases or phosphatases [37]. After that, the Rab proteins recruit motor adaptors to enhance vesicle transport along the cytoskeletal tracts (actin filaments or microtubules) [38]. When the vesicle cargos are transported to the destination membrane, Rab proteins mediate vesicle tethering by recruiting tethering factors that interact with molecules on the acceptor membrane. Those factors promote membrane fusion through their interaction with soluble N-ethylmaleimide-sensitive factor attachment protein receptors (SNAREs) [39].

Precise control of endocytic and exocytic trafficking of receptors and their ligands is essential in the regulation of cellular signaling pathways, which impact gene expression and biological functions. Since Rab25 is involved in the control of vesicle trafficking and signaling pathways, it is frequently reported to have altered expression in cancer [40, 41]. Rab25 associated protein, FIP1C, was also reported to alter cancer progression [42]. Rab25 coordinated the recycling of α5β1 integrin vesicles and the activation of various signaling pathways.

#### Down-stream effectors of Rab25

**Figure 2:**
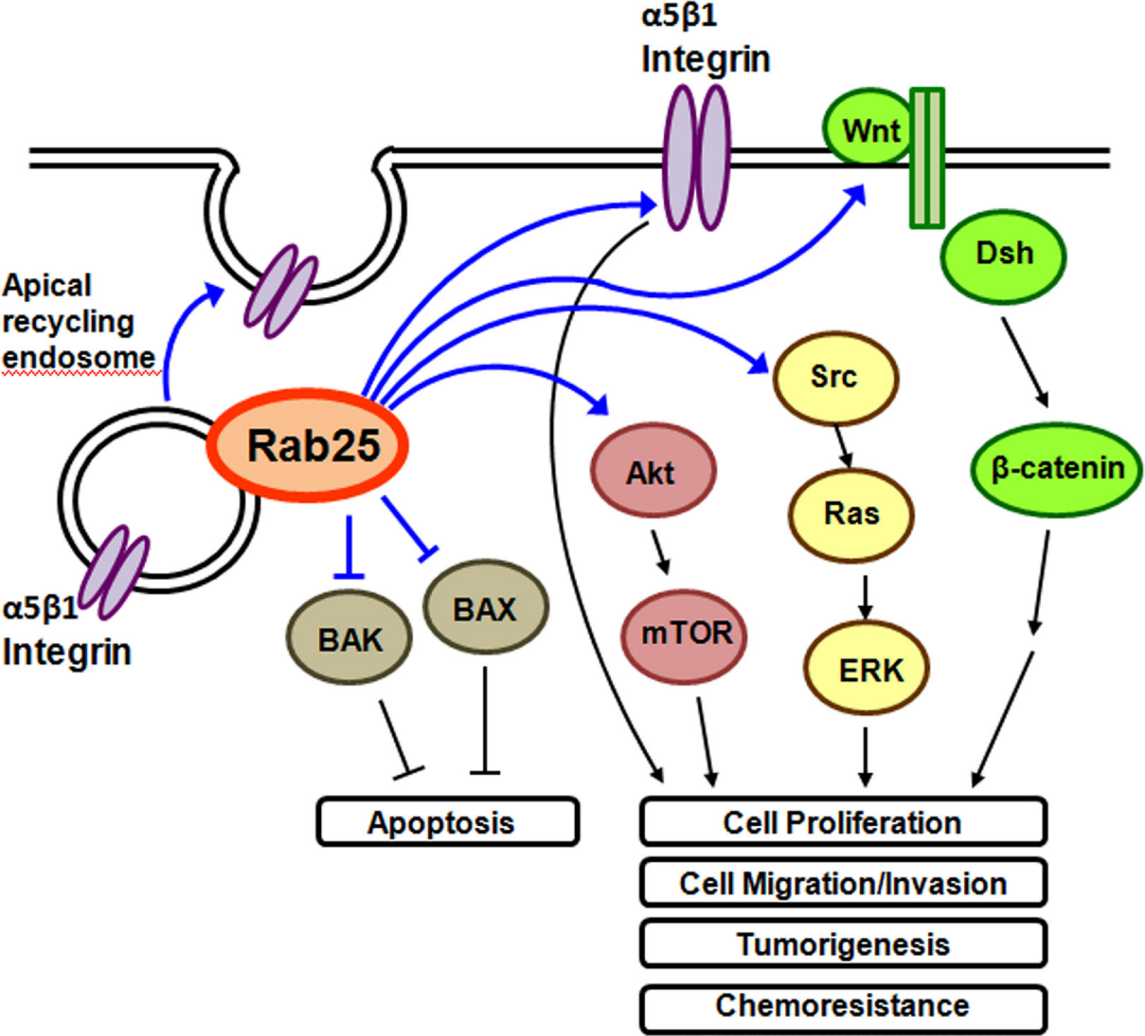
Overview of Rab25 functions in the cells Rab25 regulates apical recycling of α5β1 integrin containing endosome. Suppressive effect of Rab25 on BAX and BAK leads to reduction of apoptotic cell death. Direct Rab25 / α5β1 integrin interaction and Rab25 induced activation of Akt, Src and Wnt pathways are associated with cell growth, metastasis and chemoresistance of cancer cells.

Current evidence suggested that Rab25 is predominantly an oncogene. It promotes oncogenic functions through the regulation of integrin recycling and intracellular signaling pathways (Figure 2). Direct interaction between Rab25 and α5β1 integrin was linked to increase tumor cell metastasis and aggressiveness [43]. Modified Rab25, which does not interact with β1 integrin, did not promote cell invasion [43]. Active recycling of α5β1 integrin containing vesicle to the plasma membrane in the presence of Rab25 strongly promotes cell migration/invasion through 3D matrices [43]. Rab25 contributes to tumor progression by directing the localization of integrin-recycling vesicles and thereby enhancing the ability of tumor cells to invade the extracellular matrix. Nevertheless, it was found that Rab25 regulates integrin recycling no matter when it acts as an oncogene or a tumor suppressor. In the study of colorectal cancer, Rab25 acts as a tumor suppressor gene. Rab25-deficient mice have more tumor formation and less β1 integrin recycled to the lateral membrane [44]. However, whether suppression of β1 integrin recycling contributed to reduction of tumor formation in Rab25-deficient mice and the mechanisms involved have to be further investigated.

Rab25 activates intracellular signaling pathways, including Akt, Wnt and Src pathways and suppresses apoptotic pathway. Ectopic overexpression of Rab25 increased phospho-Akt level, which may mediate the cell proliferation-stimulating effect of Rab25 on ovarian cancer cells [12]. Rab25 knockdown reduced phospho-Akt level, leading to the reduction of cell migration/invasion in bladder cancer cell [20], hepatocellular carcinoma cells [45] and glioblastoma multiforme cells [21]. Rab25 knockdown or Akt inhibitor was found to reduce cisplatin resistance in ovarian cancer cells [46]. Rab25 was reported to activate Wnt pathway. Depletion of Rab25 inhibited the expressions of Wnt pathway target genes, including cyclin D1, c-Myc and MMP7 in hepatocellular carcinoma [45]. Rab25 promoted the expression of CLIC3, which in turn activated Src signaling pathway [47]. Ectopic overexpression of Rab25 induced phospho-ERK1/2, which is one of the downstream signaling molecules of Src, and may lead to cisplatin resistance of ovarian cancer [48]. Knockdown of Rab25 reduced phospho-ERK1/2 level and promoted cell proliferation in breast cancer [49]. Moreover, Rab25 suppressed apoptotic cell death by reducing the expression of pro-apoptotic molecules, BAX and BAK, in ovarian cancer [12]. Knockdown of Rab25 was found to reduce anti-apoptotic protein, Bcl-2, in tobacco carcinogen-induced lung cancer [50]. Taken together, activation of growth-stimulating signaling pathways and suppression of apoptotic cell death pathways mediate the effect of Rab25 on enhancing cancer progression.

#### Up-stream regulators of Rab25

Altered expression of Rab25 is commonly observed in various types of cancer. However, the mechanisms leading to Rab25 expression alteration are not fully understood. Recently, altered Rab25 expression was reported to be associated with changes in microRNA expression, Rab25 gene copy number and epigenetic regulation.

Elevated expression of several Rabs is due to downregulation of miRNAs. For example, up-regulation of Rab34 in gastric cancer is related to decreased expression of miR-9 [51]. Increase of a series of Rab proteins, including Rab21, Rab23, Rab18 and Rab3B, is due to reduction of miR-200b in breast cancer [52]. Li *et al*. has showed that Rab25 is a direct target of let-7d, which is a down-regulated miRNA in renal cell carcinoma tissues as compared with the corresponding non-tumor tissues [16].

Copy number amplification is another cause of Rab25 up-regulation. Rab25 is encoded by *RAB25* gene located at chromosome 1q22. The minimum region of recurrent amplification at the 1q22 amplicon containing *RAB25* was found in 54% of advanced serous epithelial ovarian cancers by high-resolution array comparative genomic hybridization profiling [12]. Analysis of 21 epithelial ovarian cancers showed a direct relationship between copy number and expression of Rab25, indicating mRNA overexpression of Rab25 is likely due to amplification of *RAB25* gene [12].

The expression of Rab25 has been reported to be controlled by epigenetic regulation. Rab25, an oncogene of ovarian cancer, shows a negative correlation between promoter methylation and mRNA expression [53]. In the study of ovarian cancer TCGA data set, decrease in Rab25 promoter methylation was associated with increase in Rab25 mRNA level, suggesting the elevation of Rab25 level by epigenetic regulation [53]. DNA methylation inhibitor was reported to induce Rab25 expression. The induction effect was further increased by co-treatment of DNA methylation inhibitor and histone deacetylases inhibitor in breast cancer cell [54] and lung squamous adenocarcinoma cells [55], proofing the elevation of Rab25 level by promoter demethylation and acetylation. Epigenetic regulation also led to down-regulation of Rab25 in oral and oropharyngeal squamous cell carcinoma (OOSCC) and esophageal squamous cell carcinoma (ESCC), in which Rab25 acts as a tumor suppressor. Methylation status of Rab25 promoter was significantly higher and correlated with lower Rab25 mRNA levels in OOSCC by analyzing 147 OOSCC samples in TCGA database [26]. In ESCC, demethylation treatment and bisulfite genomic sequencing analyses revealed that down-regulation of Rab25 expression in both ESCC cell lines and clinical samples was associated with promoter hypermethylation [25]. Up till now, the cause of hypermethylation of Rab25 promoter when Rab25 acts as a tumor suppressor, and vice versa, remains elusive. Further investigation is needed to understand the epigenetic regulation mechanisms of Rab25.

### Function of Rab25

#### Oncogenic function of Rab25

Rab25 is recognized to play a crucial role in tumorigenesis and cancer progression [56]. Rab25 was reported to initiate cancer formation, promote cell proliferation, suppress apoptotic cell death, enhance cell migration/invasion and increase drug resistance upon chemotherapy of various types of cancer [40]. The oncogenic function of Rab25 is likely due to its function on regulating vesicle trafficking. Rab25 increases integrin recycling to the plasma membrane and stimulating intracellular signaling pathways, which in turn regulates the oncogenic functions [41].

### Tumorigenesis

Recent reports indicated that Rab25 is related to transformed phenotype of neoplastic cells. Overexpression of Rab25 in non-transformed rat intestinal epithelial cells led to morphological and physiological transformation according to Lapierre *et al*.'s report [57]. The transformed cells changed from circular to more spindle-shaped. They were found to grow in soft agar and formed tumors in immunocompromised mice. The transformed cells also demonstrated disruption of integrin-based focal adhesions and alteration in modified microtubule subsets, indicating the transformed phenotype is microtubule-dependent. Treatment with nocodazole, a microtubule polymerization inhibitor, reversed the transformed morphology and internalization of α5β1-integrin [57].

### Cell growth/death and tumor development

It has been demonstrated that Rab25 enhances cell proliferation and tumor development. Knockdown of Rab25 expression by siRNA / shRNA transfection inhibited *in vitro* cell growth of renal cell carcinoma cells (786-O and A-498) [16], prostate cancer cells (LNCaP) [22] and hepatocellular carcinoma cells (Bel7402 and SK-Hep-1) [45]. Suppression of both *in vitro* cell growth and *in vivo* xenograft development were observed after knockdown of Rab25 in glioblastoma multiforme cells (U87MG) [21] and breast cancer cells (MCF7) [58]. Knockdown of Rab25 was reported to inhibit tumor growth in tobacco carcinogen-induced lung cancer model [50]. Conversely, ectopic overexpression of Rab25 was found to induce *in vitro* cell growth of breast cancer cells (MCF7) [12, 58] and ovarian cancer cells (A2780, DOV13, HEY, OCC1) [12]. Rab25 ectopic overexpression was found to increase development of xenograft derived from breast cancer cells (MCF7) [58] and ovarian cancer cells (A2780, HEY) [12, 59].

In addition to promote cell growth, Rab25 inhibited apoptotic and autophagic cell death. Elevated expression of Rab25 decreased UV-induced apoptosis in ovarian cancer [12]. Knockdown of Rab25 was reported to increase apoptosis in ovarian cancer cells [60] and tobacco carcinogen-induced lung cancer model [50]. Moreover, Rab25 down-regulation was reported to induce autophagic cell death in ovarian cancer from two independent research studies [60, 61]. The ability of Rab25 in cell growth stimulation and cell death suppression implicates the important role of Rab25 in supporting tumor growth.

### Cell migration/invasion and tumor metastasis

Current reports indicated the involvement of Rab25 in promoting cell migration and invasion. Knockdown of Rab25 suppressed *in vitro* cell migration and cell invasion in renal cell carcinoma cells [16], advanced non-small cell lung cancer cells [19], prostate cancer cells [22] and glioblastoma multiforme cells [21]. Suppression of cell invasion after Rab25 knockdown was observed in gastric cancer cells [17]. The finding was further supported by Geng *et al*.'s study, which showed that Rab25 depletion negatively regulated the invasion ability of hepatocellular carcinoma cells [45]. In the study of bladder cancer, Rab25 knockdown not only suppress *in vitro* cell migration but also reduce *in vivo* tumor metastasis [20]. Tail vein injection of Rab25 knockdown bladder cancer cells (EJ, T24) into immune-compromised mice has resulted in less tumor nodule formation in lung compared with the mice injected with parental bladder cancer cells [20]. Rab25 is, therefore, important in determining the metastatic ability of cancer cells.

### Chemoresistance

Rab25 induces chemoresistance towards cisplatin, a first-line chemotherapeutic agent, commonly used in the treatment of patients with various types of cancer, such as advanced non-small cell lung cancer patients [62, 63], metastatic breast cancer [64] and ovarian cancer [65]. However, cisplatin resistance limits the efficacy of chemotherapy in cancer patients [66, 67]. Rab25 expression level was significantly higher in cisplatin-resistance A549 NSCLC cells when compared with the cisplatin-sensitive A549 cells [19]. Knockdown of Rab25 by siRNA transfection decreased cisplatin resistant of NSCLC cells [19] and ovarian cancer cells [46]. On the contrary, ectopic overexpression of Rab25 in A2780 ovarian cancer cells increased cisplatin resistance in *in vitro* cell culture [48] and *in vivo* i.p. tumor xenograft [59], indicating the functional role of Rab25 in inducing cisplatin resistance in conventional chemotherapy.

### Tumor suppressor function of Rab25

Rab25 has tumor suppressor function in several types of cancer, including colorectal cancer, esophageal squamous cell carcinoma and head and neck squamous cell carcinoma [23–25]. Loss of Rab25 in human colon cancers was associated with poorer patient prognosis [44]. Rab25 deficiency promotes intestinal/colon adenoma formation in ApcMin/+ mice [44]. The result was further supported by another Rab25 deficiency study in colon cancer [68]. Rab25 knockdown Caco2-BBE colorectal cancer cells demonstrated twofold increase in the number of soft agar colonies formation and a significant increase in colony size than the control cell line [68]. Ectopic overexpression of Rab25 in esophageal squamous cell carcinoma inhibited *in vivo* xenograft tumor development and angiogenesis [25]. In the study of head and neck squamous cell carcinoma, Rab25 ectopic overexpression reduced *in vitro* cell invasion and *in vivo* tumor metastasis to cervical lymph node [24].

### Dual character of Rab25 in cancer

Rab25 has been reported to both enhance and suppress cancer progression. The role of Rab25 is cancer type-dependent. However, the mechanisms that lead to the discrepancies of Rab25 function remain elusive. One of the hypotheses suggested that the role of Rab25 depends on its ability to induce chloride intracellular channel protein 3 (CLIC3), which is involved in α5β1 integrin trafficking necessary for cancer cell invasion [47]. Pancreatic cancer patients with high Rab25 and high CLIC3 levels were associated with significantly shorter survival time. Conversely, patients with high Rab25 level and low CLIC3 levels predicted better clinical outcomes [47]. It is suggested that in the presence of CLIC3, Rab25 acts as an oncogene, whereas, in the absence of CLIC3, Rab25 acts as a tumor suppressor. When Rab25 acts as an oncogene, Rab25 enhances α5β1 integrin recycling to the plasma membrane, leading to increase in cancer progression of ovarian cancer [43]. When Rab25 acts as a tumor suppressor, Rab25-deficiency reduced β1 integrin recycling to the plasma membrane and decreased tumor formation in colorectal adenocarcinomas [44].

### Future research direction

#### Clinical implication of Rab25 in cancer

A large majority of clinicopathological findings indicate the oncogenic role of Rab25 in cancer. Elevated expression of Rab25 was significantly associated with shorter survival time of patients with bladder cancer [20], advanced non-small cell lung cancer [19], ovarian cancer [12], breast cancer [12, 49], clear cell renal cell carcinoma [15] and prostate cancer [22]. It is indicated that Rab25 is a potential prognostic marker for patients with various types of cancer.

Rab25 is up-regulated among the 52 Rab GTPases analyzed in renal cell carcinoma (RCC) according to Li *et al*.'s study [16]. High Rab25 expression level was significantly correlated with high invasion classification, lymph-node metastasis and pathological stage in RCC [16]. Moreover, high level of Rab25 expression was associated with lymph node/distant metastasis in bladder cancer [20], prostate cancer [22], gastric cancer [17] and hepatocellular carcinoma [45], suggesting the involvement of Rab25 in promoting tumor metastasis. The role or Rab25 in promoting cancer metastasis is confirmed by *in vitro* experiments in which knockdown of Rab25 decreased cell migration and invasion of 786-O and A-498 RCC cells [16].

Rab25 expression was associated with response rate of cisplatin-based chemotherapy of advanced non-small cell lung cancer (NSCLC) patients. The cisplatin-based chemotherapy response rate of NSCLC patients with Rab25-positive expression was 30%, as opposed to 52% for patients with Rab25-negative expression [19].

Elevated expression of Rab25 may be due to amplification of *RAB25* gene located in chromosome 1q22. Amplification of *RAB25* was reported to associate with markedly decreased disease-free survival or overall survival in ovarian cancer in Cheng *et al*.'s study [12]. Their report indicated an increase (at least 1.3-fold) in DNA copy number in a 1.1-Mb region located on chromosome 1q22 (which contain *RAB25* gene) in 28 of 52 (54%) of advanced serous epithelial ovarian cancers. Ovarian cancer patients with elevated *RAB25* amplification either did not enter a disease free state following surgery and chemotherapy or showed very short disease-free survival, implicating *RAB25* as potential driver gene correlated with poor prognosis and aggressive behavior of ovarian cancer [12].

Rab25 is predominantly an oncogene, but it also acts as tumor suppressor in certain types of cancer. Lower expression of Rab25 correlated with significantly shorter survival time of patients with colorectal adenocarcinomas [44] and esophageal squamous cell carcinoma (ESCC) [25]. In the study of oral and oropharyngeal squamous cell carcinoma (OOSCC), lower Rab25 expression was associated with lymph node metastasis status [26].

### Conclusion and future research direction

Rab25 GTPase regulates crucial biological functions and is important in maintaining proper function of cells. Biological and clinicopathological findings revealed that alteration of Rab25 level has high impact on cancer progression and patient survival. Rab25 may act as an oncogene or a tumor suppressor.

Rab25 overexpression and gene copy number amplification was reported in various types of cancer. Elevated expression of Rab25 correlated with poor prognosis and tumor metastatic potential of cancer patients, suggesting Rab25 is a potential marker for cancer progression. Rab25 enhances cell growth, cell migration/invasion, chemoresistance and *in vivo* tumor growth. The mechanisms for Rab25 to regulate oncogenic functions involved the control of extracellular receptors recycling and intracellular signaling pathways, including Akt, Wnt, Src and apoptotic pathways. Further investigation on the mechanisms for Rab25 mediated oncogenic functions is important for the development of novel therapeutic strategies or inhibitors targeting Rab25. Moreover, it is important to identify the mechanisms that lead to copy number amplification of *RAB25* in order to find out the potential methods for cancer prevention.

The function of Rab25 differs greatly in different types of cancer. The mechanisms that lead to the tumor suppressing function of Rab25 have not been fully understood. CLIC3 expression level was reported to be an important factor in determining the function of Rab25. In pancreatic cancer, Rab25 acts as an oncogene in the presence of CLIC3, whereas, Rab25 acts as a tumor suppressor in the absence of CLIC3 [47]. The correlation of Rab25/CLIC3 expression and clinical outcome in different types of cancer has to be investigated. In addition, the downstream mechanisms activated by Rab25 when it acts as an oncogene or a tumor suppressor are not yet clear. When Rab25 acts as an oncogene, Rab25 enhances α5β1 integrin recycling to the plasma membrane, leading to increase in cancer progression of ovarian cancer [43]. When Rab25 acts as a tumor suppressor, Rab25-deficiency reduced β1 integrin recycling to the plasma membrane and decreased tumor formation in colorectal adenocarcinomas [44]. In-depth study is needed to find out the downstream effectors that mediate the distinct function of Rab25 in cancer.

Taken together, better understanding of Rab25 function under different circumstances, together with development of Rab25 specific targeting agents, will be required for development of novel therapeutic strategy targeting Rab25 in cancer treatment.
